# A new species of the genus *Euxaldar* Fennah, 1978 from China (Hemiptera, Fulgoroidea, Issidae)

**DOI:** 10.3897/zookeys.781.27059

**Published:** 2018-08-13

**Authors:** Zheng-Guang Zhang, Zhi-Min Chang, Xiang-Sheng Chen

**Affiliations:** 1 School of Life Sciences, Jinggangshan University, Ji’an, Jiangxi, 343009, PR China Jinggangshan University Ji’an China; 2 Institute of Entomology, Guizhou University, Guiyang, Guizhou Province 550025, PR China Guizhou University Guiyang China

**Keywords:** Fulgoromorpha, Guangxi Province, Hemisphaeriini, new species

## Abstract

A new species *Euxaldarguangxiensis***sp. n.** is described and illustrated from southeastern China. The generic characteristics are redefined. A checklist and key to the species of the genus *Euxaldar* are provided.

## Introduction

The genus *Euxaldar* was erected by Fennah (1978) for a single species *E.jehucal* Fennah, 1978, described from Ninh Binh Province in Northern Vietnam (Fennah 1978). Recently this species was also recorded from Ha Noi, Vinh Phuc, Hoa Binh, and Haiphong Provinces; photos of the holotype of *E.jehucal* were provided (Gnezdilov and Constant 2012). The genus *Euxaldar* was previously placed in the tribe Issini Spinola, 1839 of the subfamily Issinae (Gnezdilov 2013). Recently, Wang et al. (2016) moved it to the tribe Hemisphaeriini Melichar, 1906 according to molecular phylogeny of Issidae. Gnezdilov et al. (2017) redescribed the type species of the genus, *E.jehucal*, and described one more species, *E.lenis* Gnezdilov, Bourgoin & Wang, 2017, from southern Vietnam. In this paper, one new species of the genus *Euxaldar* is described and illustrated from southeastern China, the generic characteristics are redefined and a checklist and key to the known species of the genus are provided.

## Materials and methods

The morphological terminology of the head and body follows Gnezdilov, Bourgoin and Wang (2017), and the terminology of male genitalia follows Gnezdilov (2003). The genital segments of the examined specimens were macerated in 10% KOH and drawn from preparations in glycerin jelly using a light microscope. Photographs of the specimens were made using Zeiss stereo Discovery V8. Microscope with Zeiss Axio Cam HRc camera, images were produced using the software Helicon Focus ver.6.7 and Photoshop CS4.0. The holotype of the new species is deposited in School of Life Sciences, Jinggangshan University, China.

## Taxonomy

### Family Issidae Spinola, 1839

#### Subfamily Hemisphaeriinae Melichar, 1906

##### Tribe Hemisphaeriini Melichar, 1906

###### Genus Euxaldar

Taxon classificationAnimaliaHemipteraIssidae

Fennah, 1978

Euxaldar Fennah, 1978: 267.

####### Type species.

*Euxaldarjehucal* Fennah, 1978, by monotypy.

####### Diagnosis.

Body hemispherical, head including eyes wider than pronotum. Metope flat and elongate. Coryphe transverse, 2-3 times as wide as long. Fore wings elongate and wide, without hypocostal plate; venation poorly recognizable. Hind wings one-lobed, rudimentary, much shorter than fore wings. Hind tibia with two lateral spines. First metatarsomere with two latero-apical spines and 6-7 intermediate spines. Gonoplacs rounded. Phallobase asymmetrical, narrow, with basal or subapical processes; ventral phalobase lobe shorter than the dorsal lobe. Aedeagus without ventral hooks. Male anal tube enlarged apically or elongate (in dorsal view).

**Figures 1–11.**
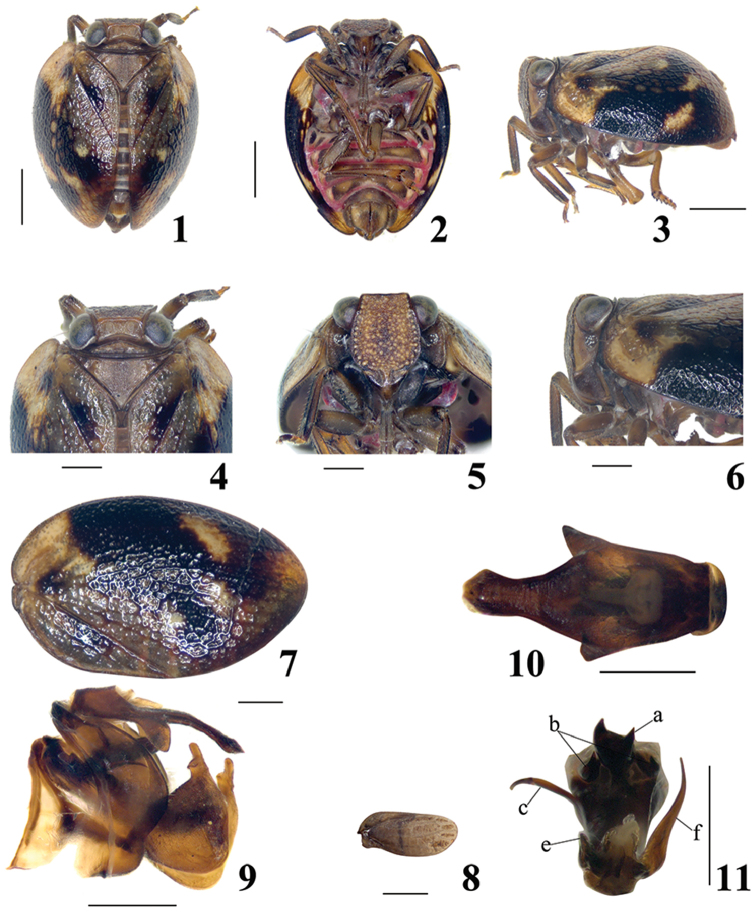
*E.guangxiensis* sp. n. **1** Adult (male), dorsal view **2** Adult (male), in ventral view **3** Adult (male), in lateral view **4** Head and thorax (male), in dorsal view **5** Face (male), in frontal view **6** Head (male), in lateral view **7**Fore wing (male) **8** Hind wing (male) **9** Male genitalia, in lateral view **10** Anal tube, in dorsal view **11** Penis, in dorsal view from caudad; Scale bars: 1.0 mm (**1–3**), 0.5 mm (**4–11**).

**Figures 12–20.**
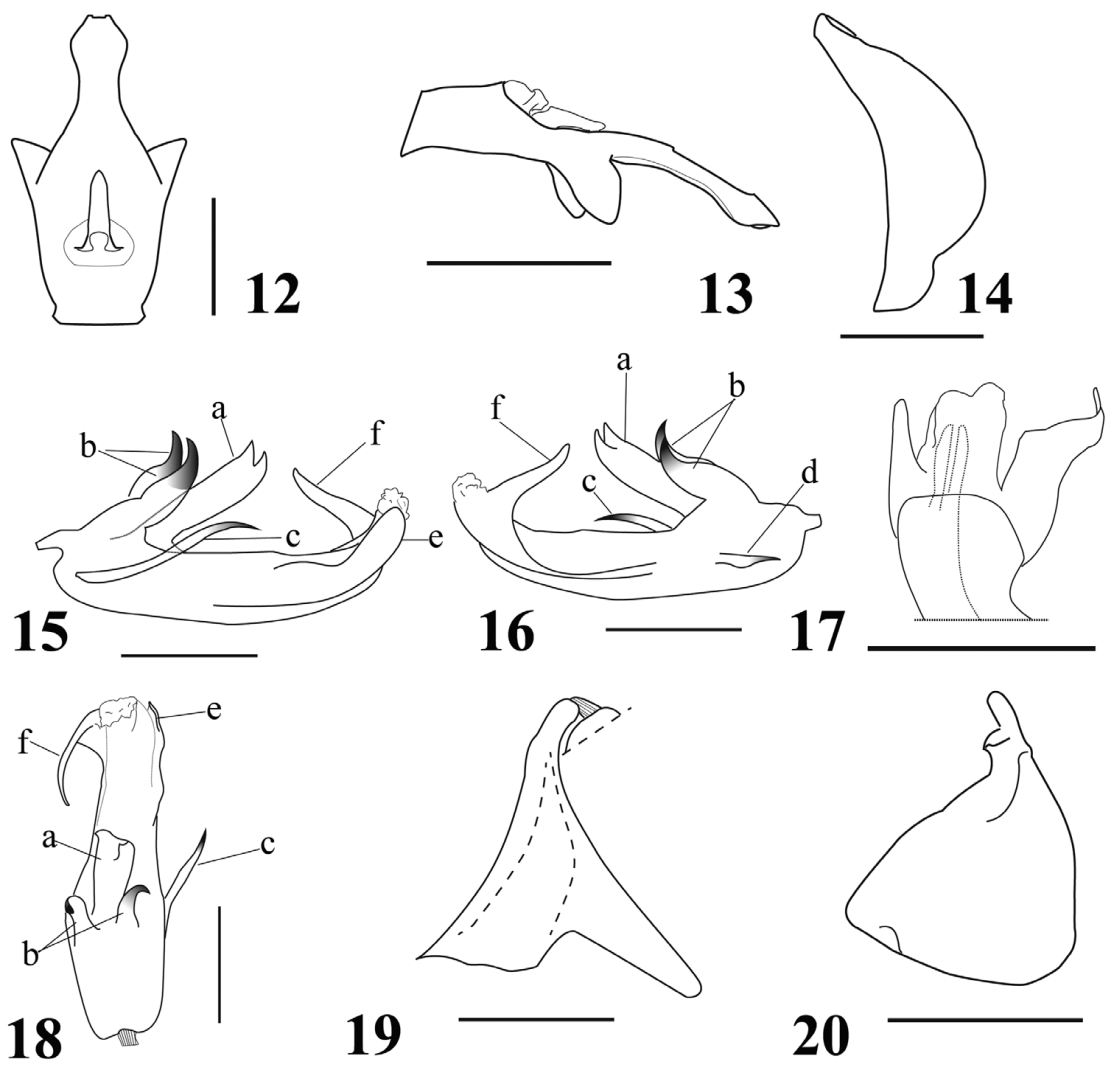
*E.guangxiensis* sp. n. **12** Anal tube (male), in dorsal view **13** Anal tube (male), in lateral view **14** Pygofer (male), in lateral view **15** Penis, in lateral view (left) **16** Penis, in lateral view (right) **17** Penis, in ventral view **18** Penis, in dorsal view **19** Connective, in lateral view **20** Gonostylus, in lateral view. Scale bars: 0.5 mm.

####### Distribution.

**Figure 21.**
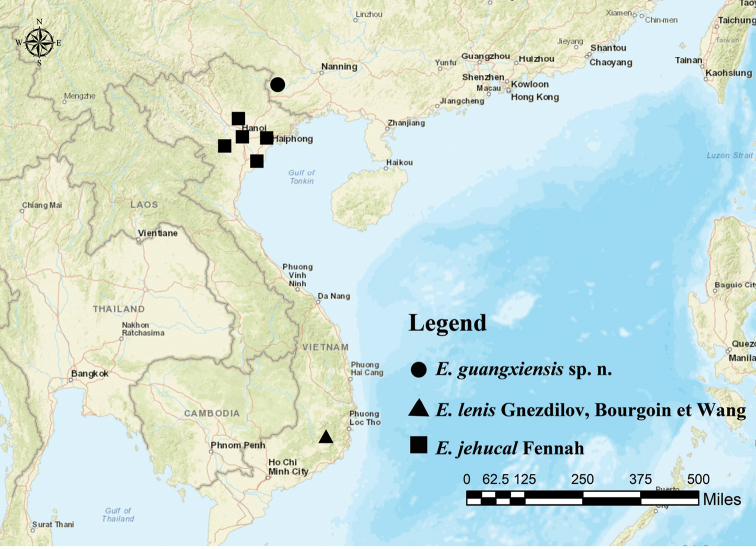
Geographic distribution of *Euxaldar* species.

China (Guangxi); Vietnam (Ninh Binh, Ha Noi, Vinh Phuc, Hoa Binh, Haiphong and Lam Dong Provinces) (Figure 21).

####### List of *Euxaldar* species

*E.jehucal* Fennah, 1978 (Vietnam: Ninh Binh, Ha Noi, Vinh Phuc, Hoa Binh and Haiphong Provinces)

*E.lenis* Gnezdilov, Bourgoin & Wang, 2017 (Vietnam: Lam Dong Provinces)

*E.guangxiensis* sp. n. (China: Guangxi Province)

##### Key to species of the genus *Euxaldar* modified from Gnezdilov et al. 2017

**Table:** 

1	Metope smooth, without any pustules (Gnezdilov et al. 2017: fig. 23)	***E.lenis* Gnezdilov, Bourgoin & Wang**
–	Metope with row of distinct pustules along its lateral margins (Fig. 5; Gnezdilov et al. 2017: fig. 20)	**2**
2	Metopoclypeal suture complete. Male anal tube deeply concave posteromedially (in dorsal view) (Gnezdilov et al. 2017: fig. 6)	***E.jehucal* Fennah**
–	Metopoclypeal suture incomplete medially (Fig. 5). Male anal tube elongate, wide at base, narrow at apical part, laterally with two triangular processes near middle part (Figs 10, 12–13)	***E.guangxiensis* sp. n.**

###### Euxaldar guangxiensis sp. n.

Taxon classificationAnimaliaHemipteraIssidae

http://zoobank.org/D77A38F8-F9C9-423C-9FF3-E30F4E30EABF

Figs 1–11 , 12–20

####### Type material.

Holotype: 1 ♂, China: Guangxi, Nonggang National Nature Reserve (E106°58'3", N22°28'37"), 163 m, 29 Oct. 2017, K.K. Liu

####### Description.

Body length (from apex of vertex to tip of forewing): male 3.8mm; Forewing: male 3.3mm

**Coloration.** Male: Coryphe (Figure 4) dark brown. Metope light brown yellowish, with pale pustules along its lateral margins. Clypeus (Figure 5) pale brown with dark brown band at base, rostrum and antenna dark brown (Figure 5). Pronotum and mesonotum brown (Figure 4). Forewings (Figure 7) dark brown, each with wide black band at midlength from costal margin to almost apex of clavus and with several light yellow patches including large one in basal part of the wing. Hind wing (Figure 8) dark brown. Legs (Figs 2–3) brown with dark brown markings. Abdomen (Figure 2) dark brown, with margins rufous.

**Head and thorax.** Coryphe (Figure 4) transverse, approximately 3.0 times wider than long, without carinae, anterior margin nearly straight, posterior margin slightly angularly concave. Metope (Figure 5) flat, 1.1 times longer than widest, without a median carina, with a row of distinct pustules along its lateral margins and rather weak pustules inside. Metopoclypeal suture (Figure 5) incomplete medially. Postclypeus with wide median carina. Pronotum (Figure 4) short, with keel-shaped margins. Paradiscal fields very narrow behind the eyes. Mesonotum (Figure 4) 3.3 times longer than pronotum in midline, with lateral carinae. Fore wings (Figure 7) oval, with smoothed, poorly recognizable reticulate venation; CuP distinct. Hind wings (Figure 8) rudimentary, 0.3 times as long as fore wings, veins obscure. Hind tibiae with 2 lateral teeth near apex. Spinal formula of the hind leg 7-7-2.

**Male genitalia.** Anal tube (Figs 10, 12, 13) elongate, wide at base part and narrow at apical part, slightly enlarged near apex, apical margin concave medially, laterally with two triangular processes near its middle. Anal column (Figure 12) located near base, 0.3 times as long as the anal tube in dorsal view. Pygofer (Figs 9, 14) in lateral view, with posterior margin distinctly convex. Phallobase asymmetrical, dorsally with three processes at base (Figure 18a, b), middle process of phallobase (Figs 15–16a, 18a) wide, with two teeth apically, lateral processes of phallobase (Figs 15, 16b, 18b) adjacent to middle process hook-shaped. Phallobase laterally with two processes near base, one of them is long and directed caudally (Figure 15c), the other short and directed cephalad (Figure 16d). Lateral phallobase lobes asymmetrical, narrowing apically, one is short directed caudally (Figure 15e), the other is long and curved cephalically (Figs 15f, 16f). Ventral phallobase lobe (Figure 17) not reaching the aedeagal apex, apical margin nearly straight. Connective (Figure 19) in shape of long and narrow cup. Gonostylus (Figure 20) triangular, with moderately convex hind margin, caudo-dorsal angle widely rounded.

####### Etymology.

The specific name refers to the locality, Guangxi province, China.

####### Host plant.

Unknown.

####### Distribution.

China (Guangxi province)

####### Remarks.

This species resembles *E.jehucal* and *E.lenis*, but can be distinguished from the latter in the following characteristics: Anal tube (Figs 10, 12–13) longer than broad, narrowing from half to apex, slightly expanded near apex, apical margin concave medially, laterally with triangular processes; phallobase (Figure 18) with three processes at base in dorsal view, middle process wide (Figs 15, 16a, 18a), with two teeth apically, lateral processes (Figs 15, 16b, 18b) hook-shaped; phallobase laterally with two processes (Figs 15c, 16d); lateral phallobase lobes asymmetrical, narrowing apically, one is short (Figure 15e), the other is long and curved cephalad (Figs 15f, 16f).

## Discussion

The genus *Euxaldar* is similar to *Neohemisphaerius* Chen, Zhang & Chang, 2014, but differs as follows: Posterior margin of coryphe slightly angularly concave (Figure 4); Metope slightly longer in midline than widest, median carinae absent (Figure 5); Metope and clypeus joint at nearly right angle (Figure 6); Clypeus without hump-like processes (Figure 5); Aedeagus without ventral hooks (Figs 15, 16); *Neohemisphaerius*: Posterior margin of coryphe obviously angularly concave (see Chen et al. 2014: figs 2–35C, 2–36C; Zhang et al. 2016: fig. 1); Metope elongate, distinctly longer in midline than widest, median carinae obviously present (see Chen et al. 2014: figs 2–35E, 2–36E; Zhang et al. 2016: figs 3, 6); Metope and clypeus joint at nearly obtuse angle (see Chen et al. 2014: figs 2–35D, 2–36D; Zhang et al. 2016: figs 2, 5); Clypeus with a hump-like process medially (see Chen et al. 2014: figs 2–35E, 2–36E; Zhang et al. 2016: figs 3, 6); Aedeagus with pair of ventral hooks (see Chen et al. 2014: figs 2–35M, 2–36L; Zhang et al. 2016: fig. 9).

## Supplementary Material

XML Treatment for Euxaldar

XML Treatment for Euxaldar guangxiensis
